# Ritual morphospace revisited: the form, function and factor structure of ritual practice

**DOI:** 10.1098/rstb.2019.0436

**Published:** 2020-06-29

**Authors:** Rohan Kapitány, Christopher Kavanagh, Harvey Whitehouse

**Affiliations:** 1 School of Psychology, Keele University, Keele, Staffordshire, UK; 2 School of Anthropology and Museum Ethnography, University of Oxford, Oxford, UK; 3 Department of Psychology, Rikkyo University, Tokyo, Japan

**Keywords:** ritual, cross-cultural, emotion, anthropology, ritual modes, cultural evolution

## Abstract

Human rituals exhibit bewildering diversity, from the Mauritian Kavadi to Catholic communion. Is this diversity infinitely plastic or are there some general dimensions along which ritual features vary? We analyse two cross-cultural datasets: one drawn from the anthropological record and another novel contemporary dataset, to examine whether a consistent underlying set of latent dimensions in ritual structure and experiences can be detected. First, we conduct a factor analysis on 651 rituals from 74 cultural groups, in which 102 binary variables are coded. We find a reliable set of dimensions emerged, which provide potential candidates for foundational elements of ritual form. Notably, we find that the expression of features associated with dysphoric and euphoric experiences in rituals appears to be largely orthogonal. Second, we follow-up with a pre-registered factor analysis examining contemporary ritual experiences of 779 individuals from Japan, India and the US. We find supporting evidence that ritual experiences are clustered in relatively orthogonal euphoric, dysphoric, frequency and cognitive dimensions. Our findings suggest that there are important regularities in the diversity of ritual expression and experience observed across both time and culture. We discuss the implications of these findings for cognitive theories of ritual and cultural evolution.

This article is part of the theme issue ‘Ritual renaissance: new insights into the most human of behaviours'.

## 1. Introduction

Social scientists, historians and archaeologists have recorded a great diversity of human rituals from around the world. And in so doing, they have identified features common across rituals, such as stereotypy, repetition, causal opacity, goal demotion, normative prescriptions and orthodoxy [1–10]. But to what extent do these aspects of ritual behaviour vary in a systematic way cross-culturally, and how robust is the dimensionality of ritual generally? Here, we present two studies that seek to address whether similar underlying factors of ritual emerge from two distinct contexts: first, a coded database of ethnographic ritual accounts, and second, a contemporary dataset of ritual experiences collected from 779 respondents in India, Japan and the US. In Study 1, we conducted a theoretically agnostic factor analysis on an existing database of 651 rituals from 74 cultural groups. In Study 2—informed by the results obtained in Study 1, which broadly conformed to the ‘Modes of religiosity’ theoretical framework [4]—we restricted our pre-registered factor analyses to four key factors, examining whether they emerge from a survey of contemporary ritual experiences collected from individuals in India, Japan and the US.

In using a factor analysis, there is no expectation that the results will produce a sensible factor structure, so our primary research question was whether the latent variables extracted would be distributed randomly or, as we expect, form clusters that can be interpreted sensibly in light of existing theories. Based on the results of Study 1, in Study 2 we aimed to address whether the factors extracted accord with those outlined specifically in Modes theory [4]. Our two studies address different time periods (recent history and the contemporary era) and approach ritual experiences at different levels of analysis, with Study 1 focusing on the analysis of second-order coding of recorded ritual accounts and Study 2 focusing on self-reported ritual experiences. This increases the potential that different factors will emerge between the two studies but may also provide converging evidence reflecting core recurrent aspects of ritual morphology.

## 2. Ritual in the context of Tinbergen's four questions

We understand ritual to be a special category of social action that (a) includes predefined sequences of action characterized by rigidity, formality and repetition, which is (b) embedded in systems of meaning and symbolism, and which (c) contains non-instrumental elements (i.e. causally opaque and goal demoted elements) [9]. A full discussion of alternative definitions is beyond the scope of the present article, but the definitions we employ here (and most other definitions available) describe ritual at a level beyond that of concrete features. What we hope to do is to begin to describe the ‘morphospace’ of ritual form, which we construe as analogous to the phenotypic features common to the ritual ‘family’ of behaviour, in which many diverse expressions exist within respective ‘genera’ and ‘species’.^1^ In so doing, we hope to provide broader foundations upon which further empirical study can address the ontogeny, phylogeny, mechanism and adaptive features of ritual.

The level of coherence in the category of ritual is an issue that remains under dispute. Some scholars regard the category of ritual as too broad to be useful; see for instance Boyer & Liénard [11], who argue that ‘Ritual … is used to denote disparate forms of behavior, on the basis of a faint family resemblance’. Others, however, suggest that there is a typology [12] and shared psychological components [9]. These are issues that are empirically tractable and upon which our present investigation can shed some light. In principle, a description of ritual focusing on the phenomenological morphology allows a more fine-grained and complementary analysis of what ritual may be, the role it plays and how it emerges. For example, also in this issue, Nielsen *et al*. [13] discuss ritual and ritualized action among *Homo neanderthalensis*, specifically addressing the phylogenetic and adaptive qualities of the phenomenon in the prehistoric record. But theirs, and indeed anyone else's, discussion of the ontogeny and life-history of ritual—which in this case relate to an early emerging capacity for ritual cognition via the expression of over-imitation [14] and the ritual stance [8,15–17]—must be clear on what the concrete dimensions of the phenomena are.

## 3. Ritual morphology and theories of ritual form

While the range of possible forms that ritual could take is potentially limitless, the forms that actually stabilize and are passed down through the generations in cultural traditions are shaped and constrained by features of human cognition, including intuitive biases, memory capacities and emotional systems [3,6,7,18]. Rituals are also constrained in various ways by the demands of wider social environments in which they form, often performing useful social functions ranging from the regulation of family life to the integration of entire political systems [19,20]. As a result of these shaping and constraining factors, rituals that survive and flourish in cultural traditions tend to cluster around discernible attractor positions in the morphospace of all possible ritual forms.

There are several well-developed theories of ritual form, including ‘Modes of religiosity’ [4], McCauley & Lawson's [21] ‘Ritual form hypothesis' and Schjoedt *et al*.'s ‘Cognitive resource depletion’ model [22], as well as anthropological theories associated with *collective effervescence* [23] and *communitas* [24]. Consider that McCauley & Lawson [21] argue that rituals are structured in such a way that agents and patients (respectively, those executing the ritual, and those being acted on by a ritual), objects and actions must be delineated from ordinary people, objects and actions by the way of ‘S-markers’ indicating supernatural efficacy. For example, some rituals can only be correctly performed by a priest or a shaman who is supernaturally empowered to act; one cannot simply swear on a book, one must swear on a holy book because of its supernatural properties. Our data ought to reveal whether cultural rituals require such special status items: that is, the latent factors that emerge might constitute specific categories of S-marked features, or, a singular S-marker factor might emerge in which multiple S-marked objects load. Meanwhile, classic perspectives in anthropology suggest that rituals ought to generate ‘*collective effervescence*’ [23], which may be recognizable by the way of social, physical or psychological pageantry; factor analyses may reveal dimensions corresponding with one, or more, ‘pageantry’ factors.

‘Modes of religiosity’ is perhaps the most empirically well-described theory to date, and it makes a structural claim about ritual form: rituals will trend towards high-arousal, low-frequency morphologies (imagistic) or low-arousal, high-frequency morphologies (doctrinal) [4]. The former occurs infrequently (paradigmatically only once a lifetime, or once a generation) and are often intensely dysphoric (e.g. arousing considerable negative affect via painful or terrifying experiences). Doctrinal rituals, by contrast, are very frequent (sometimes occurring many times a day or a week and at least as often as multiple times a year); they are also less emotionally intense than imagistic rituals and may even be quite tedious. This predicted ritual structure, however, is probabilistic rather than law-like and so—while many confirmatory examples exist—there are notable exceptions. Consider, for example, Pentecostal Christian traditions, which are both (euphorically) intense and highly frequent [25], while divinatory rituals [26], which may involve infrequent participation, arouse only low emotional responses. Thus, the Modes theory of ritual form tends to describe two ‘attractor positions’ which account for ritual form at the aggregate level.

Atkinson & Whitehouse [5] curated a database of 651 rituals from the ethnographic record which were coded for 102 variables. The database was specifically designed to test the Modes theory but also coded for the presence or absence of over 100 other variables qualities). Correlational analysis found that ritual frequency is negatively correlated with measures of ‘arousal’ (*ρ* = −0.40) and that the relationship is stronger for dysphoric (*ρ* = −0.41) than euphoric experiences (*ρ* = −0.08).^2^ The data suggest two distinct relationships between dysphoric and euphoric arousal and frequency. Broadly speaking, there is an incremental increase in dysphoric arousal for each reduction interval in frequency, with the rate of increase holding steady over daily, monthly, seasonal and annual ritual events, and thereafter roughly doubling for less-than-annual and doubling again for once-in-a-generation rituals. Euphoric arousal, on the other hand, displays a similar increase as frequency reduces from daily to annual rituals, but after that, the trend reverses and arousal scores decrease for less-than-annual and generational rituals. Overall, the relationship between emotional arousal and frequency was consistent, with the most infrequent events, such as initiations, displaying highest arousal scores (for both euphoric and dysphoric dimensions), while more frequent rituals demonstrated lower arousal scores [5, p. 55].

## 4. Study 1. A high-resolution examination of ritual modes in the anthropological record

In Study 1, we re-analysed the dataset created by Atkinson & Whitehouse [5] based on data from 74 cultures extracted from the electronic Human Relations Area Files (eHRAF) database. These rituals were drawn from the ‘Probability Sample Files' (PSF), which have been specifically curated to avoid issues of non-independence in the data [27]. For full details on the nature of the data, see Atkinson & Whitehouse [5]. Each ritual was coded as belonging to one of 17 categories (a list is available in electronic supplementary material, table S9), and a total of 102 elements were coded for whether they were present/absent based on the anthropological record. Using only these binary values, we conducted a factor analysis to determine whether there is an apparent set of dimensions of ritual form that emerges from the cross-cultural ritual dataset.

### (a) Analysis

Factor analyses of binary data^3^ require a different approach from for continuous or ordinal data. Best practices and simulation studies show that polychoric matrices are most appropriate, as they produce the most accurate measures of correlation and loading values, but unavoidably produce suboptimal measures of fit [29–31]; equamax rotations maximize correct grouping of binary variables and minimize incorrect grouping [32]. Thus, we executed a factor analysis using a polychoric correlation matrix [29,31,33] with equamax rotation with a loading threshold of 0.4. We attempted recommended factor extraction rules [34,35], but of all attempted analyses, these rules produced values that had the worst fit and explained the least variance (see [36,37]).

We analysed all data using R. Each of the 651 entries was randomly allocated to either an Exploratory or Confirmatory analysis dataset, a process known as cross-validation [38]. In total, 325 rituals constituted the exploratory factor analysis (EFA) dataset, while 326 constituted the confirmatory factor analysis (CFA) dataset. In some cases, instances of reported behaviour were low in the complete dataset, and the randomization process created some variables that had zero-frequency in one of the randomized sets. For the sake of analytic assumptions, any variable that had zero-frequencies were omitted from both datasets. Within the data presented here, six variables were omitted (the number in parentheses is how many times these features showed up in the complete dataset): short duration (*n* = 0), scarification (*n* = 1), sucking of patient (*n* = 3), disgust (*n* = 1), human sacrifice (*n* = 4) and a smoking taboo (*n* = 2). Both the EFA and CFA dataset contained 96 common features of ritual.^4^

Our approach identifies whether variables reliably load onto similar factors across the EFA and CFA, thus revealing whether the items have ‘stability’ [36]. However, given that the dataset was not compiled for this analytical technique explicitly, we conduct additional analyses to rule out other interpretations.

### (b) Results

**Figure 1.**
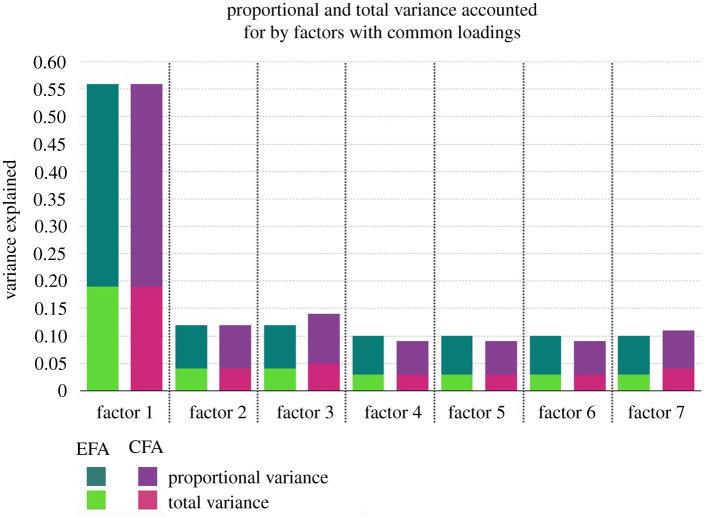
Proportional and total variance accounted for by factors with common loadings. Note: values in this figure are stacked, not superimposed. Thus, the total variance explained by factor 1 in the EFA is 0.19, and the proportion of the variance explained in the model is 0.37 (not 0.56).

The EFA suggested an 11-factor solution that explained 0.50 cumulative variance, in which the root-mean-square residual (RMSR) was 0.06 (lower than the 0.08 threshold), and the root-mean-square error of approximation (RMSEA) was 0.117 (greater than the 0.08 threshold). The CFA was constrained to 11 factors. It explained 0.51 cumulative variance, in which the RMSR was 0.06, and the RMSEA was 0.118. While each model contained 11 factors, only the first seven had items common to both analyses. Figure 1 shows the proportional and total variance explained in both the EFA and CFA. Table 1 reveals which items were common and their respective loading values (statistics for factors 8–11, and item loadings for all items, are reported in electronic supplementary material A). We conducted a range of follow-up analyses (available in electronic supplementary material B) in which we tested for factor structure of (i) simulated datasets and (ii) random samples of the present dataset, by (iii) mode/frequency of the ritual, and (iv) whether or not the factor structure was a consequence of the over-representation of a particular category of ritual. The present model vastly outperformed all alternatives.

**Table 1.** Factors and item loadings for items that loaded in common on both EFA and CFA datasets. Note: factor 1 had a total of 37 items common to the factor in both datasets (see electronic supplementary material, table S1); for brevity's sake, we list the 10 with the highest loadings in each dataset. All other factors are complete.

factor 1	factor 2	factor 3	factor 4	factor 5	factor 6	factor 7
‘dysphoric elements’	‘euphoric elements’	‘pageantry—physical’	‘viscera’	‘pageantry—psychological’	‘frequency’	‘kin’
other (catch-all) negative, humiliation,	average euphoria (0.70; 0.75)	dancing (0.83; 0.61)	blood (0.59; 0.66)	burning offerings (0.51; 0.51)	annual participation (0.69; 0.69)	extended kin (0.63; 0.72)
the burning of participants,						
tattooing, vomiting,	peak euphoria (0.66; 0.82)	percussion (0.72; 0.68)	animal sacrifice (0.71; 0.63)	fire embers (0.50; 0.47)	annual (as patient) (0.68; 0.64)	immediate kin (0.72; 0.45)
laceration of sensitive areas,						
mental ordeals, piercing,		intense music/dance (0.46; 0.53)		stimulants (0.48; 0.54)		
swallowing of objects (not eating),						
large-scale music and dance		singing (0.46; 0.65)		hallucinogens (0.47; 0.54)		

### (c) Discussion

We find support for the claim that rituals have a coherent underlying dimensionality as the factors extracted are, for the most part, interpretable. The extraction of dysphoric and euphoric factors and the amount of variance they accord for is in line with ritual theories that focus on emotional arousal and pageantry, especially Modes theory, or those that focus on costly signals [22,39]. Factor 1 contains items that arouse negative affect and are consistently dysphoric (and accounts for 0.19 of total variance explained), while factor 2 (which accounts for 0.04 of total variance) contains items related to positive affect that are generally euphoric in nature. Factor 3 items—dancing, singing, percussion and intense music—are neither clearly positive nor negative and may suggest that ‘pageantry’ does not skew valence in either direction but instead may serve as an intensity multiplier. Factor 5 appears to describe a kind of psychological pageantry associated with inebriation and altered states of consciousness—drug use and fire use are speculated to have played an important role in prehistoric rituals [40,41]. Factor 4, the ‘viscera’ factor, explains around 0.03 variance and is somewhat like the pageantry factor in that is, *a priori*, neither clearly positive nor negative. Factor 7, which explains around 0.04 variance, relates exclusively to kin; here, it is notable that group size variables (*2 or 3 people, fewer than 15 people, less than half the community, more than half the community, and most of the community*) did not load on this factor.

We also note that some variables of theoretical interest did not appear to reliably load on any given factor (or weakly cross-load in any meaningful way), specifically elements that relate to semantic information or explicit symbolism, such as the recitation of text(s), speechmaking and the presence of holy texts or holy objects. This does not count as disconfirming evidence of McCauley & Lawson's hypotheses that rituals require S-markers; however, our data provide no support for this claim. We note, however, that only a few such special item variables were included in the database and, therefore, avoid drawing any strong conclusions.

The factor analysis suggests that negative and euphoric elements are not oppositional but orthogonal within rituals (the correlation within each ritual for intensity scores on *peak* euphoria and peak dysphoria across all rituals *r* = 0.01, n.s.; while the correlation within each ritual between *overall* euphoria and overall dysphoria is *r* = −0.16, *p* < 0.001). This analysis indicates that while there is a weak negative correlation, rituals may contain both dysphoric and euphoric elements and that it is the relative proportions from each factor that may produce dysphoric or euphoric rituals. A wedding, for example, is frequently described by its participants and patients as being positive, though the participants also describe the experience of the event as provoking anxiety. Similarly, even dysphoric rituals (such as hazings or initiations) eventually give way to relief and may be experienced, or evaluated in hindsight, as positive [42]. All of this is to illustrate the point that positive and negative affect can coexist *within* a ritual and may, in fact, become more pronounced *after* the ritual as individuals reflect on their experience [42]. Atkinson & Whitehouse [5] demonstrated negative correlations between emotional arousal and frequency, but as previously discussed, this relationship varied between euphoric and dysphoric arousal *overall*. Taken together, these findings suggest that any model that attempts to quantify rituals as expressed along a single axis of emotional arousal (where dysphoric and euphoric affect are treated as oppositional poles) is underspecified. This relates to an ongoing debate among emotion researchers (see [43–45]) as to whether negative and positive affect are bipolar or independent [46,47]. Our results align with the summary presented by Schimmack [48] that positive and negative affect ‘are clearly separable components of affective wellbeing, although they may not be strictly independent’ [49, p. 113].

Our analysis suggests the presence of seven factors, which broadly accord with prominent theories related to the dimensionality of ritual form. It was possible, prior to analysis, that several alternative structures might have been revealed (see electronic supplementary material B for additional alternative analyses). One was that euphoric and dysphoric elements would negatively load on the same factor (which would support a unitary, bipolar conception of arousal). A second possibility was that each factor might generally correspond with a given ritual type, such as a wedding-factor, a funeral-factor, and an initiation-factor. Third, it was also possible that such an analysis might not reveal any sensible structure, and that dimensionality and morphotype of rituals are relatively unconstrained and arbitrary. Instead, we observed distinctive valence factors, and factors that correspond to physical and psychological pageantry. Alongside their correspondence with recent cognitive models of ritual, these factors also relate to classic anthropological frameworks that focus on rituals' ability to generate *collective effervescence* [23] or *communitas* [24]. The viscera and kin factors are intriguing but do not immediately appear to correspond with existing frameworks.

Overall, our findings suggest that the form of ritual cross-culturally is canalized by cognitive and potentially environmental and social constraints and that, therefore, the ‘morphospace’ of ritual form is constrained along meaningful joints, including notably dysphoric elements and euphoric elements, as well as physical and psychological pageantry. While we acknowledge that factor solutions are imposed structures and may not necessarily correspond with the ‘true’ structure of the phenomenon, we suggest that the empirically revealed factors, including those of ‘pageantry’, ‘viscera’ and ‘kin’ ought to be considered worthy of future investigation (weighted for their relative contribution to the overall model), and we encourage other authors to incorporate these dimensions of the ritual structure in future theorizing.

## 5. Study 2. An examination of contemporary ritual accounts

Study 1 examined the ritual structure using an unconstrained factor analysis that identified seven components among ethnographic accounts of ritual morphology. In Study 2, we applied a similar analysis to contemporary accounts of collective ritual experiences in three countries: the US, India and Japan. These countries were selected as they are geographically and culturally distinct, with rituals that revolve predominantly around Christian, Hindu and Buddhist/Shinto traditions, respectively. Pragmatically, they also have urban populations that are easily accessible via online data collection platforms. The samples represent a mix of population contexts, with the US participants providing a mix of non-religious and religious, Indian participants predominantly representing Hinduism, and Japanese participants demonstrating an orthopraxic mix of non-religious and Buddhist/Shinto. Although there is inevitably some overlap and cultural cross-fertilization between these countries, including in seasonal ritual celebrations (e.g. Diwali in the US and Christmas in Japan), we anticipated that broadly they represent a diverse collection of ritual environments. Moreover, we concur with the anthropologist Roy Rappaport's declaration that ‘no society is devoid of what a reasonable observer would recognize as ritual’ [50, p. 31].

Sampling from such diverse environments we collected self-generated collective ritual accounts and asked people to rate the experiences they described on a variety of self-assessment metrics (described below). To increase the diversity of experiences described and to capture both infrequent and common rituals we asked participants the same sets of questions in relation to (1) the most memorable collective ritual they had experienced and (2) the collective ritual they perform most frequently. We focused on *collective* rituals to try and decrease the amount of idiosyncratic personal routines described.

Given the findings of Study 1, the significant existing empirical work on Modes theory and the framing of our ritual prompts around frequent and memorable rituals, in this study, we imposed an anticipated structure on our factor analysis specifying four factors that accord with the theoretical model of Modes theory but also relate to the ‘Cognitive resource depletion in religious interactions’ [22] and McCauley & Lawson's ‘Ritual form hypothesis’ [21]. We pre-registered our expectations for the factors that would emerge, which we categorized as follows: (i) dysphoric (negative affect), (ii) euphoric (positive affect), (iii) frequency, and (iv) cognitive (a factor pertaining to semantic knowledge or ritual exegesis in line with the prominent theories of ritual cognition indicated above). In line with Study 1, we expected euphoric elements to load onto a separate factor from dysphoric elements, rather than to load in an oppositional manner on the same factor.

### (a) Methods

Study 2 was pre-registered. We aimed to collect data from at least 200 individuals in three different countries—the US, Japan and India (for a total minimum *N* = 600) to serve as a contemporary dataset that we could compare with the ethnographic data examined in Study 1. Participants were recruited on Amazon Mechanical Turk (US/India) and lancers.co.jp (Japan) which are both online crowdsourcing platforms in which anonymous participants complete short tasks for payment. Participants were asked to provide demographic information and were prompted to define—in their own terms—what a ‘ritual’ is, and to self-generate a list of five rituals. We then asked participants to report on two of their own ritual experiences: (1) the most memorable *collective* ritual they had experienced and (2) the *collective* ritual they perform most frequently.^5^ We avoided any reference as to the emotional valence of the ritual experiences, but our questions did weakly orientate responses along an axis of frequency (although, notably, we did not prescribe that the memorable ritual ought to be infrequent or intense). Participants then provided ratings of each experience on 27 focal variables, including items that related to procedural details, emotional response, frequency of performance and levels of reflection. All quantitative measures were collected on a 100-point sliding scale (full methods and pre-registration file are available in electronic supplementary material C).

### (b) Results

Three datasets (one each from the US, Japan and India) were collected. We over-sampled, anticipating the need to remove participants who failed to complete the task in sufficient detail. In each location respectively, a total of 417 (the US), 378 (Japan) and 339 (India) individuals began the survey. The cleaning process was the same for each dataset. First, participants who did not complete at least 85% of the survey were removed (total participants removed for insufficient data, the US: 104; Japan: 94 and India: 88), then participants who completed the survey in less than 10 min were removed (the US: 15, Japan: 0 and India: 54; the expected duration of the survey was 25 min). After removal of the fastest participants, the mean duration in the US was 27.73 min; in Japan, it was 31.05 min; and in India, it was 35.58 min. The final dataset contained 779 individuals (the US: 298, Japan: 284 and India: 197). The US dataset contained 162 females and 134 males (2 undisclosed), and the mean age was 35.8 years. The Japan dataset contained 142 females and 141 males (1 undisclosed), and the mean age was 39.1 years. The India dataset contained 60 females and 132 males (5 undisclosed), and the mean age was 32.3 years.

We used attention checks not as an exclusion criterion but as an indicator that the responses should be checked for the quality of responses, as previous research suggests they are an unreliable indicator of overall quality. In the US, 78 (of 298) failed a single attention check; in Japan, 54 (of 284) failed a single attention check; and in India, 48 (of 197) failed both attention checks,^6^ and 90 failed one of the two attention checks. We examined whether those who failed the attention check varied from those who passed in systematic ways. Thus, we categorized all responses as belonging to either the imagistic or doctrinal modes per the question prompt. We conducted an ANOVA on the following key variables for each mode, within each country: *intensity*, whether the ritual was considered *routine*, how *consequential* the ritual was to the participant, how many *times participated* in, and how well the participant *remembered* the experience. We used a corrected *p*-value of 0.003 for these 15 analyses (as there are five analyses for each ‘mode’ for each country; though results do not change with an uncorrected value). Full results are available in electronic supplementary material D; suffice to say here that we found no differences among the US and Indian responses, respectively, and only one difference among Japanese responses (the ‘routine’ variable was significantly different between modes). Given that there was no systematic pattern to the observed differences between those who passed and failed attention checks, we opted not to exclude participants (who surpassed more basic exclusion criteria).

**Figure 2.**
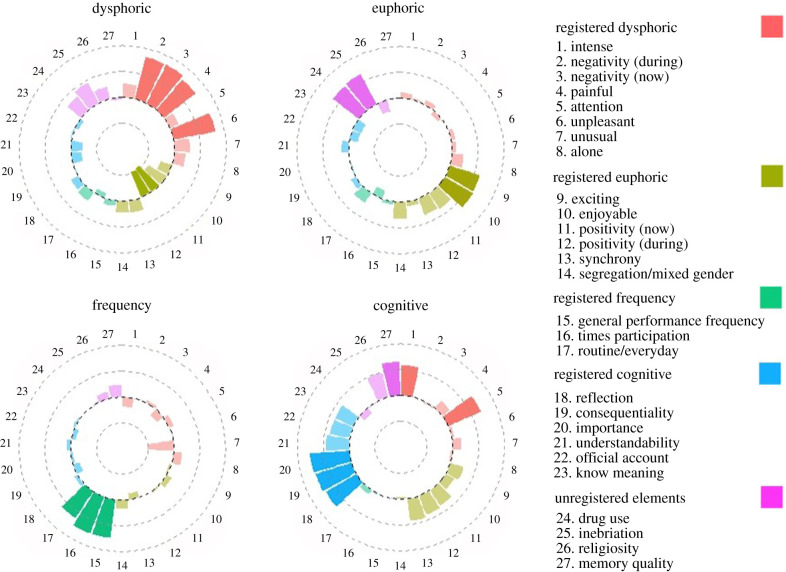
The revealed and predicted factor structure and item loadings. Note: the dysphoric factor explained 0.16 variance, the euphoric factor explained 0.09 variance, the frequency factor explained 0.16 variance, and the cognitive factor explained 0.08 variance.

Per our pre-registration (https://osf.io/m7hca), we constrained our analyses to four factors using an ‘oblimin’ rotation. Figure 2 shows the loadings of the 27 focal variables, and on which factor they loaded. Darker colours loaded above the threshold of 0.5 (or below −0.5) and can be regarded as the revealed factor structure (a complete list of factor loadings are available in electronic supplementary material, table S12 and electronic supplementary material E). A parallel analysis indicated the appropriateness of a four-factor model as there were four values above the 1.0 threshold; *χ*^2^ = 3893.85, *p* < 0.001. The RMSEA = 0.096 (90% CI: 0.092–0.098), while the RMSR = 0.04. The model explains 0.50 total variance overall. The euphoric and cognitive factors correlate at 0.39, with all other correlations less than 0.17 (absolute).

Our pre-registered hypotheses were largely supported; however, contrary to our prediction, participants' evaluation of positivity (during the event and after the event, respectively, ‘then’ and ‘now’) negatively loaded on the dysphoric factors. Despite this, an independent euphoric factor still emerged (with drug use and inebriation also loading strongly; see electronic supplementary material, table S12 for comprehensive values). The predicted frequency factor was observed, as was a cognitive factor, with the expected additional loading of memory quality. Intensity and attention also co-loaded on the cognitive factor and not on the dysphoric factor as predicted. While we in no way are making a *post hoc* claim to the prescience of this, these loadings are interpretable within the current findings. As with Study 1, we note that ‘religiosity’ did not reliably load on any factor, and, somewhat contrary to Study 1, drug use and inebriation loaded on the euphoric factor.

### (c) Discussion

We pre-registered the prediction that four factors would emerge; we predicted a euphoric and a dysphoric factor, as well as a frequency factor, in line with the findings from Study 1 and Modes theory; we also anticipated a cognitive factor based on modes and other prominent theories of ritual cognition (see [21,22]). Study 1 suggested that viscera, kin and pageantry were relevant dimensions of ritual forms, but we did not expect this pattern to emerge in Study 2 owing to the significant distinctions in cultural settings between the small-scale tribal societies that are represented in the ethnographic data and the contemporary samples in this study being drawn from online, predominantly urban respondents. Rather, we anticipated and predicted the most empirically well-supported factors of the ritual structure to emerge. In this regard, we expected dysphoric and euphoric dimensions to emerge, as well as a dimension of frequency. We also included a fourth cognitive dimension owing to the emphasis placed on ritual reflection and meaning-making in prominent theories of the ritual structure and function, whether ‘spontaneous exegetical reflection’ or semantic doctrinal knowledge as per Modes theory [4], an ‘inferential gap’ per the cognitive depletion model of religious interactions [22].

Two points need to be made with regard to the correlation between the euphoric and dysphoric factors (*r* = 0.4). First, Study 1 used an equamax rotation—which maximizes the correct grouping of binary variables and minimizes incorrect grouping [32]—which is an orthogonal method of rotation. Based on the nature of the data used in Study 1 (collected ethnographic accounts that were systematically coded), we believe that this decision was justified. However, the change in the level of analyses between coded elements of rituals accounts and the experience of rituals at an individual level meant that such an assumption is not tenable for the data in Study 2. Consequently, in Study 2 we used an oblimin rotation which permits correlation between factors (though does not require it). The second point is that the two factors are positively correlated. Broadly put, increasing scores on ‘euphoric variables’ positively predicts increasing scores on ‘dysphoric variables’. This supports the view that both euphoric and dysphoric emotional arousal contribute in a mutually reinforcing manner to the overall level of emotional arousal of a given ritual.

## 6. General discussion

In our abstract, we posed the following question: to what extent do aspects of ritual behaviour vary in a systematic way cross-culturally, and how robust is the dimensionality of ritual generally? This is an important question as the level of variation across ‘ritual’ contexts can appear extreme, encompassing the bloody human sacrifices of the Aztecs in Mesoamerica and the intense piercing *Kavadi* rituals performed during Tamil Thaipusam festivals, alongside the solemn and subdued performance of Catholic communion and the silent daily performances of Islamic prayer (*salat*). We recognize each of these examples as ‘rituals’, but should we? Our results suggest that despite the diversity found in the expression of ritual morphology there are detectable structural features that are, at least to some degree, recurrent in both ethnographic and contemporary ritual accounts.

We show that by examining the features of a ritual (such as the presence or absence of 102 possible variables—including holy texts, fire, blood, singing and various kinds of taboos), there is a dissociable set of latent structures in which euphoric, dysphoric and frequency-related elements aggregate with other similar variables. But we also show that there are dimensions of both physical and psychological pageantry which are related with classical anthropological ritual frameworks such as that of *collective effervescence* [23] or *communitas* [24], while dimensions associated with viscera, kin and cognition also emerge. However, we note that in both ethnographic and contemporary datasets, it was the dysphoric factor that accounted for the largest share of variation in our measures. This suggests that the emphasis placed on dysphoric rituals by Modes theory, costly signalling theories and ‘Automatic accrual’ theory is warranted [39,51,52]. Our results indicate that neither geography nor ritual function (such as marriages, funerals and initiations) provides a coherent explanation of the factor structure observed.

There have been many previous attempts to categorize rituals and many such efforts have focused on their social and psychological functions. Our findings do not contradict the utility of such frameworks, but rather indicate that in future exploration of rituals, it is important to give due consideration to the factors identified in Study 1. This is not, however, to suggest that each of the factors identified will be equally relevant in all contexts. As the results of Study 2 demonstrate, restricting dimensionality to a smaller number of factors can still produce meaningful outcomes. Our recommendation, therefore, is that researchers consider the dimensions relevant to the rituals they are examining. We do however recommend that across all contexts researchers should distinguish both dysphoric and euphoric features of ritual events (Study 1) and euphoric and dysphoric dimensions of *subjective* responses to the ritual experiences (Study 2). Our results, in line with other recent examinations of dysphoric ritual events, indicate that this is a crucial distinction even if positive and negative affect interact positively to establish overall emotional arousal ([5]; 51, ch. 8).

However, the data examined are not without limitations, and these are important to acknowledge. In Study 1, the presence or absence of a given feature was extracted from the anthropological record. In the past, anthropologists typically did not record what was absent (save for unique instances in which absence was conspicuous, say, a wedding in which a bride was absent), while some features may have been present but not recorded, depending on the depth and breadth of the anthropologists' research interests and the relevance of cultural features to their goals (e.g. if a fire was present for instrumental or tangential reasons, it may not have been recorded). Thus, Study 1 should be regarded as an agglomeration of *salient* features of ritual, acknowledging that the absence of a feature may not correspond with a real absence, but rather its diminutive or tangential role in the target of investigation. Moreover, the method of analysis in Study 1 (known as cross-validation [38]) involves randomly splitting a large dataset into two parts: ‘exploring’ possible models in one dataset and ‘confirming’ in the other. This terminology is misleading; we prefer to conceive of the findings as ‘reliable’, and given that we were looking for commonality of variables in specific factors across the two datasets (rather than attempting to clearly delineate variance accounted for) we recommend a cautious interpretation. Given also the size of the dataset, randomly splitting the data in half can produce datasets of unequal size, which may require cleaning (i.e. the removing of variables from both datasets if a variable has no instances in one of the two datasets), and as such, results may vary over iterations. Again, this leads us to recommend a cautious interpretation and to regard the present results as requiring further verification. Additionally, while we claim that factor analysis is agnostic to theory, it is the case that the dataset we used was originally compiled to test hypotheses associated with Modes theory and this likely skewed the variables examined. That said, given that 102 variables were coded, we judge the list of features examined to be overall quite comprehensive, but invite interested scholars to examine the relevant items and judge for themselves if any significant feature was overlooked.

Additional limitations are apparent for Study 2. Data were collected from a total of 779 individuals from Japan, the US and India—across 27 variables—and were analysed according to a pre-registered plan. We acknowledge that there was a degree of correlation between the factors and that the explicitly euphoric factors (positive ‘then’ and ‘now’) also loaded negatively onto the dysphoric factor (though the ‘euphoric’ elements still better constituted a separate ‘euphoric’ factor). It is also possible that the factor structure observed is not unique to ritual contexts, but rather a function of the specific items measured: that is, it could be argued that there are euphoric, dysphoric, frequency and cognitive dimensions to most experiences. We acknowledge this issue and encourage ritual researchers, when possible, to examine theoretical models in non-ritual ‘control’ contexts. We recognize this to be an aspect missing from the current paper, but hope that our dataset may be useful as a comparison for other researchers. We also note that while rituals represent an interesting category of social action, it is not the case that they are entirely distinct from all other kinds of social action, indeed rituals are often embedded in larger social contexts. Yet despite this, there are important distinctions and we have no reason, for example, to imagine that the emotional intensity of a ritualistic ballet performance of *Swan Lake* should be inversely related to its frequency of performance. Nor do we have good theoretical motivations to anticipate that *Swan Lake* performances will generate predictable responses in terms of reflection and group bonding. Thus, although the dimensions identified may represent a generalized set that relates to social action broadly, we argue that they apply more specifically to ritual settings.

Future direction of this line of enquiry may involve examining exactly how rituals—either at the level of individual rituals or the level of ritual category—cluster within the morphospace. Herein, we have shown that there is likely dimensionality to rituals, but analytical work mirrors that of [54], who demonstrated not only how four categories of songs (dance, healing, love and lullaby) common to 86 cultures exhibit a reliable structure along three dimensions (analogously corresponding to the seven dimensions of ritual presented here), but that those songs cluster in possibility space in such a way that they are commonly closer to same-category songs than songs from other categories. We have laid the groundwork for such analyses, and hope that more tailored datasets can be generated, and such analyses may be conducted.

In conclusion, our results acknowledge the existence of dimensions of ritual structure—dysphoric and euphoric arousal, physical and psychological pageantry, viscera and kin—which provides us with a better understanding of the ‘morphospace’ of ritual form. Having provided this tool, we hope that researchers may be better equipped to focus on addressing questions of ritual ontogeny, phylogeny, mechanism and adaptiveness in a more calibrated manner. Put another way, we hope to have offered a complementary diagnostic tool that describes the features of ritual at the taxonomic level of the ‘family’, allowing for a diversity and categorization of ritual expressions at the analogous levels of ‘genus’ and ‘species’. Our data and code are made public for reviewer and reader inspection (https://osf.io/undx8/).

## Supplementary Material

Supplementary Materials for 'Ritual Morphospace Revisted'

## Endnote

1 We caution against an overly literal interpretation of this analogy, as culture and biology do not have a perfect correspondence. However, inasmuch as we might recognize that rituals within the islamic tradition share commonalities, or that wedding rituals from diverse faiths also share commonalities, the analogy is conditionally apt.

2 All results discussed are significant unless otherwise indicated.

3 Two instances of continuous data were coerced into a binary form. The four-level ‘duration’ variable (short, medium, long and extended) was dichotomized into single variables—we noted no ‘short’ values, and so we created three mutually exclusive binary variables (e.g. medium = 1/0; long = 1/0 and extended = 1/0). ‘Typical frequency as patient’ and ‘… as participant’ (seven levels) was recorded under ‘occurring once a year or less frequently’ (1), or ‘more often than once a year’ (0). The four measures of *average* and *peak* positive and negative arousal were recorded in a continuous/ordinal format, which we modified to simply reflect whether or not the *average* and *peak* arousal were either more euphoric (1) or dysphoric (0). All empty cells were imputed a ‘0’ (absent). The reasoning for this decision is that if the qualitative anthropological record failed to describe it, we can reasonably infer that it was not present, or not relevant in the judgement of expert anthropologists. Per a recent publication [28], we do not believe Laplace's indifference principle can be applied here to sensibly arrive at a value between present and absent. We omitted all qualitative data.

4 Owing to the random nature of allocation, these values may change between iterations.

5 We note that by allowing participants to select rituals based on their own understanding of the term, we ended up collecting data in which participants reported some events, like family meals or drug-taking, as rituals. The vast majority of responses describe weddings, funerals, initiations and cultural events. We have not excluded responses that seem less definitionally ritualistic, as they constitute only a minority of responses, and if the participant categorizes such actions as being ritualistic, then they likely share consequent features and cognitions associated with rituals. Our data are available for review (https://osf.io/undx8/).

6 The US and Japan designs both included only one attention check each, but the Indian sample included two.

## Data accessibility

Data and syntax are available from: https://osf.io/undx8/.
