# Supplementary material for: Ritual morphospace revisited: the form, function and factor structure of ritual practice
Source: Philos Trans R Soc Lond B Biol Sci. 2020 Jun 29;375(1805):20190436. doi: 10.1098/rstb.2019.0436 (PMC7423254; doi:10.1098/rstb.2019.0436)
Supplement: Supplementary Materials for 'Ritual Morphospace Revisted' [file rstb20190436supp1.pdf]

Supplementary Material A.

Factor 1. The first factor in the EFA and CFA accounts for .19 total variance, and .37 explained variance in each dataset. Factors in red have strong cross-loadings on other factors. Items that did not load on the first factor in both analyses are listed at the bottom. We cautiously interpret this factor as 'Dysphoria'. It is notable that the direct measures of affect do not load on this factor.

Table S1. *Variables and their respective loading values on the first factor*

**Variables**	**Loadings in EFA**	**Loadings in CFA**
Beating	0.537387	0.523305
Burial Offerings	0.419585	0.560197
Burning Offerings	0.501631	0.583328
BurningParticipants	0.857948	0.632646
Circumcision	0.568947	0.547966
Confession	0.445413	0.846747
Dehydration	0.57851	0.628271
DramaticActs	0.514238	0.438677
Fear	0.592254	0.524004
FoulOlfaction	0.51443	0.657694
sexing	0.675982	0.485921
Fumigation	0.548844	0.452134
Games	0.476922	0.445099
Hallucinogen	0.602314	0.632078
Humiliation	0.842365	0.656303
Immobility	0.523126	0.656454
InterdictionHands	0.654402	0.660126
LacerationSensitive	0.86788	0.602129
LargescaleMusicDance	0.842089	0.563039
OtherEndurance	0.542664	0.6235
OtherMental	0.743207	0.71971
OtherNegative	0.743659	0.8741

PathogenicPossession	0.677995	0.424895
Piercing	0.598808	0.857983
Sedatives	0.469605	0.542484
Smoking	0.42274	0.455729
SorrowMusicDance	0.619661	0.70544
Spitting	0.489755	0.582202
StoryTelling	0.420922	0.485574
SwallowingObjects	0.734547	0.719129
TabooAlcohol	0.642534	0.749122
TabooTalking	0.743863	0.555318
Tattooing	0.72668	0.758526
Vomiting	0.877383	0.601129
WaterOffering	0.402936	0.414733
WoundingSignificant	0.488351	0.858967
AthleticFeats	0.486627	
Haircut		0.449247
Isolation	0.40836	
Naming		0.401601
OtherMental		0.868361
PerformanceTrance	0.467208	
SleepDeprivation		0.40425
Stimulants	0.467957	
Taboo Sex	0.552072	
WoundingSuperficial	0.462373	

Note: Factors in red have strong cross-loadings on other factors.

Factor 2. The second factor in the EFA and CFA accounts for .04 total variance, and .08 explained variance in each dataset respectively. The only common items between the analysis are the average affect and peak affect. We cautiously interpret this factor as 'Euphoria'. It is notable that the direct measures of affect do not load on this factor. These affect values was coded such that if the event had higher positive than negative affect it was coded

as 1, while if it had higher negative than positive affect it was coded as 0. Thus, values positive load on this, denoting a degree of orthogonality between euphoric and dysphoric affect (as it was entirely possible that these values would negative load factor 1).

Table S2. *Variables and their respective loading values on the second factor*

Variables	Loadings in EFA	Loadings in CFA
AvPrimaryEuphoria	0.696604	0.752187
PeakPrimaryEuphoria	0.66128	0.823408
Blood	-0.43354	
Isolation	-0.7077	
WoundingSignificant	-0.42289	
Isolation		-0.45752
Joyful		0.479547

Factor 3. The third factor in the EFA accounts for .04 total variance, and .08 explained variance, and in the CFA it accounted for .05 total variance, and .09 explained variance. We tentatively interpret the third factor ‘Pageantry - Physical’.

Table 3. *Variables and their respective loading values on the third factor*

Variables	Loadings in EFA	Loadings in CFA
Dancing	0.825115	0.6147
Percussion	0.72459	0.676113
IntenseMusicDance	0.46455	0.530293
Singing	0.462103	0.65009
NonRhythmic	0.420204	
Marching		0.445074
Weapons		0.415999
Outsiders		0.402884
OtherInstruments		0.39925

Factor 4. The fourth factor in the EFA accounts for .03 total variance in each dataset, and .07 explained variance in the EFA and .06 explained variance in the CFA. We tentatively interpret the third factor 'Viscera'

Table 4. Variables and their respective loading values on the fourth factor

Variables	Loadings in EFA	Loadings in CFA
SacrificeAnimal	0.7147	0.634046
Blood	0.591472	0.664887
OtherOffering	0.584451	
Weapons		0.420103

Factor 5. The fifth factor in the EFA accounts for .03 total variance, and .07 explained variance in the EFA and .06 explained variance in the CFA. We tentatively interpret the third factor 'Pageantry - Psychological'.

Table 5. Variables and their respective loading values on the fifth factor

Variables	Loadings in EFA	Loadings in CFA
BurningOfferings	0.51334	0.512471
FireEmbers	0.50227	0.472749
Stimulants	0.477117	0.53615
Hallucinogen	0.474447	0.543029
Marching	0.398611	
OtherInstruments	0.488432	
OtherPurify		0.471566
Vomiting		0.436079

Factor 6. The sixth factor in the EFA accounts for .03 total variance, and .07 explained variance in the EFA and .06 explained variance in the CFA. We tentatively interpret the third factor 'Frequency'. A reminder that the coding for the '*Typical Frequency as patient*' and '*... as participant*' was initially recorded with 7-levels, which for analytical reasons were dichotomized, such that '*occurring once a year or less frequently*' (1), or '*more often than once a year* (0). It shouldn't be particularly surprising that both measures of frequency would load together, but what is notable is that the values did not load on the *Dysphoria* (factor 1) or *Euphoria factors* (factor 2). Modes

theory suggests that as intensity increases, frequency decreases. Thus, these frequency values could have conceivable loaded onto either, or both, of these affect measures.

Table 6. Variables and their respective loading values on the sixth factor

Variables	Loadings in EFA	Loadings in CFA
AnnualPARTICIPANT	0.693711	0.687426
AnnualPATIENT	0.679473	0.643986
Washing	0.547185	

Factor 7. The seventh factor in the EFA accounts for .03 total variance, and .07 explained variance in the EFA; the same factor in the CFA accounts for .04 total variance and .07 explained variance (likely due to the additional variables that loaded on to it). We tentatively interpret the third factor 'Kin'. Two things are noteworthy. First, the negative loadings in the EFA. The valence of the loadings are essentially arbitrary, though it is the case that both kin values aggregate together. Second, as there are fewer items accounting for diminishing variance, we are seeing less stability between the EFA and CFA.

Table 7. Variables and their respective loading values on the seventh factor

Variables	Loadings in EFA	Loadings in CFA
ExtendedKin	-0.6332	0.442471
ImmediateKin	-0.71683	0.454215
Alcohol	0.425744	
PerformanceTrance		0.729825
ExecutivePossession		0.713227
PathogenicPossession		0.558804
MixedGender		0.524344
MockConflict	0.396486	

Subsequent factors. Variables loaded onto several other factors, but did not appear to load reliably between the EFA and CFA analyses. They are presented in Table 8.

Table 8. Variables and their respective loading values on the remaining factors

Exploratory Factor Analysis		Confirmatory Factor Analysis	
MR8		MR7	
Spitting	-0.39985	TextRecitation	0.657251
OtherDiscomfort	-0.42435	BurningParticipants	0.441185
PathogenicPossession	-0.42717	Speechmaking	0.437269
Sprinkling	-0.49098	OtherOffering	0.419185
variance explained	*Total = .03; Prop = .05*	*variance explained*	*Total = .03; Prop = .06*
MR11		MR8	
LargeCrowd	0.717866	Group50percent	0.607774
Group90100percent	0.498584	WoundingSuperficial	0.474768
Group15abs	-0.59885	Group15abs	-0.58718
variance explained	*Total = .03; Prop = .05*	*variance explained*	*Total = .03; Prop = .06*
MR7		MR9	
Extended_duration	0.528699	Long_duration	0.48265
Outsiders	0.524926	Outsiders	0.412506
variance explained	*Total = .03; Prop = .05*	*variance explained*	*Total = .02; Prop = .05*
MR10		MR10	
Medium_duration	0.790248	Alcohol	0.527252
Long_duration	-0.5968	LargescaleMusicDance	0.515766
variance explained	*Total = .03; Prop = .05*	HolyObjects	0.415624
		variance explained	*Total = .02; Prop = .05*

Supplementary Material B.

Follow up analyses

The factor analysis suggests that there may be a factor structure that applies to the dataset. However, given that the dataset was not compiled for this analytical technique explicitly, additional analyses ought to be conducted in order to rule out other interpretations. Moreover, factor solutions are simply imposed structures, and may not necessarily correspond with the 'true' structure of the phenomenon. As is the case here, the EFA was conducted in such a way as to find an acceptable structure on a random-split half of a dataset, then, this n-factor structure was applied to the other matched-half. In the absence of theory, this in no way implies that the structure described corresponds with reality. We did find, however, that the structure broadly conforms with existing theory, and may also accommodate other evolutionary principles (i.e., involvement of kin).

In order to check against the most egregious violations of false structure, we generated random datasets of binary values with 651 observations and 96 variables, we found that parallel tests revealed no meaningful factor solutions, and when we forced specific factor solutions on the data, the variance explained was less than .01 per factor with fit statistics hovering around .5.

We also attempted to analyse the invariance in order to determine whether the same factor structure was present within sub-populations (category). However, doing so with binary data is difficult [52]. A simple alternative for approximating invariance, at least with regard to our specific hypotheses, is to conduct reliability analyses within the categories in question. Krippendorff's Alpha [53] is a reliability metric that can help us determine an alpha value for the present dataset. We used the *'Irr'* package for R, and present the alpha values associated with specific categories. We found that for each category (i.e., funeral, weddings, liturgies, divinations, etc.) that reliability for the 96 items ranged between .235 and .375 well below the accepted threshold of .8 [54] (see Table S9 for specific values and the number of observations).

Table S9. *The number of observations and Krippendorff's Alpha value for each category of ritual.*

**Ritual Category**	**Observations**	**Alpha**
WorldyPursuit	269	0.279
Funerary	93	0.287
OtherPurpose	70	0.271
Initiation	69	0.293
OtherWorldyPursuit	45	0.262
NewBorn	36	0.316

Apotropaeic	33	0.289
Divination	28	0.296
Commemorative	26	0.272
Wedding	21	0.314
Liturgical	18	0.291
HouseDedication	11	0.326
OtherUnion	8	0.268
Ogather	8	0.235
Protection	6	0.274
Installation	4	0.375

In order to provide a benchmark alpha, we repeatedly randomly sampled 40 rituals from the dataset 15 times and conducted Krippendorf's reliability analysis on each. These 15 values ranged from .25 to .315. Thus, the values reported for each ritual are essentially indistinguishable from randomly sampling rituals from across categories. When the dataset was crudely split by frequency, approximately corresponding with each mode (i.e., rituals that occur less often than annually, and rituals that occur more often than annually), and were matched for having the same elements, we found that low frequency ('imagistic') dataset (n = 505) and high frequency ('doctrinal') dataset (n = 140) shared 75 common elements, and had internal alphas of .239, and .324 respectively. Indeed, the EFA (n = .325) and CFA (n = .326) datasets which were originally randomly determined, and which shared 96 common variables, had internal alphas of .287 and .274, respectively.

Supplementary Material C.

Methods

The following procedure was conducted in three countries: The United States, Japan, and India. In all cases the survey was administered on a computer, and participants were solicited through Amazon MechanicalTurk (US and India), and lancers.co.jp (Japan). In the US it was presented in English. In Japan it was presented in japanese. The Japanese survey was prepared by one of the authors (CK) and then quality checked by two native Japanese researchers. In India it was presented in Hindi. A native Hindi translator produced an initial translation from the English text, and this was back-translated and differences in meaning clarified by two additional native Hindi translators. The first examined the initial translation (which led to some minor changes), and the second checked the quality of the amended translation. The English, Japanese, and Hindi translations are available on OSF.io. The survey was expected to take 20 minutes, and we paid our participants at a rate corresponding to the legal hourly rate. All participants were offered $2US per hour. The mean duration of the survey in each location was 28.85 mins, 31.08 mins, and 37.05 mins, respectively.

Participants were asked to provide demographic information, and were prompted to list five rituals, and to define - in their own terms - what a 'ritual' is. Participants were then asked to spend not more than 2 - 3 minutes describing '*the most memorable collective ritual you have experienced during your life*' and the '*the collective ritual you perform most frequently'* (participants were informed that they could not advance the survey until at least 90 seconds had elapsed; the order of these questions was randomized). Participants then provided data on the 26 focal variables (as described in the pre-registration) as well as limited number of other variables (reported in table S10). All measures were collected on a 100-point sliding scale, with five written metrics. Each was a variation on the following: the 0-anchor was 'not at all', following by 'a little', 'moderate', and 'very', and the 100-anchor was 'extremely' (or 'constantly, or 'perfectly' depending on the question). Some questions were categorical.

Because participants provided self-generated examples of rituals on which to base their responses, we examined all written responses and coded for appropriateness (for the indian and japanese responses, the responses were translated by google translate[1]). Responses the described actions that were consistent with Hobson et al's. (2018) definition were coded as '2' (e.g., weddings, funerals, graduations, etc.), responses that were broadly ritualistic by lacked the broader symbolism and doctrine were coded as '1' (e.g., football chants, japanese ritualised business meetings, formal family dinners, etc.), and responses that lacked clear ritualistic elements, but were clearly habitual (e.g., reading to one's children before bed) or highly sentimental (e.g., a couple

[1] We recognize that this tool lacks nuance in many regards, however, since we were not interested in the specifics of their response, but rather that participants responses were broadly ritualistic, we found the tool adequate.

sharing a regular 'date night') were coded as '0'. The authors RK coded these. Responses that were coded as 0 by RK were excluded from analyses (41, 7, 44 ommission from the imagistic set; 72, 22, 62 omissions from the doctrinal set). Our final analysable dataset included 491 from US (with 280 imagistic and 211 doctrinal responses), 567 from Japan (with 287 imagistic and 280 doctrinal responses), and 300 from India (with 159 imagistic and 149 doctrinal responses). All raw data and R syntax is available at (https://osf.io/undx8/).

Pre-registration and analysis plan

We made the following predictions regarding the factor structure of the data (see Table S10). All predictions were for positive loadings. We predicted four factors, using a 'direct oblimin' rotation (as we anticipated some degree of correlation between factors). We will conduct a more exploratory factor analysis in order to determine whether the data suggest a superior fit for a number of factors other than 4. We will use standard evaluative techniques, including parallel lines and eigenvalues.

We will also measure whether drug use was involved in the ritual experience (including, but not limited to, alcohol). We make no specific prediction regarding the loading of 'drugs' onto any individual factor, but suspect it will cross-load moderately across multiple factors. Similarly, measures that signify explicit religiosity (i.e., sacred objects and places) did not reliably load in prior work. Here, we predict that evaluations of 'religiousness' will cross-load on multiple factors. Finally, 'memory quality' may load on factors A and B, as the qualities of those events are linked with the production of vivid, enduring, flashbulb like events, but may load on factor D, due to the cognitive nature of the measurement. See table S11 for summary of ambiguous variables.

Table S10. *Pre-registered factors*[2].

**Factor A**<br>**Dysphoric (8)**	**Factor B**<br>**Euphoric (6)**	**Factor C**<br>**Praxis (3)**	**Factor D**<br>**Cognitive/ Doctrinal (6)**
Intense<br>Negativity (during)<br>Negativity (now)<br>Painful<br>Attention<br>Unpleasant<br>Unusual	Exciting<br>Enjoyable<br>Positivity (now)<br>Positivity (during)<br>Synchrony<br>Segregation / Mixed Gender	General performance frequency<br>Times participation<br>Routine/everyday	Reflection<br>Consequentiality<br>Importance<br>Understandability<br>~<br>Official Account<br>Know meaning

[2] We have used letters rather than numbers to describe predicted factors, as we are not strictly attempting to predict the variance explained by each factor, but simply its presence.

Alone			

We also note of the following: Factor analysis is a statistical tool that describes data that *represents a thing*, and is not *the thing itself*. Regarding Factor D, we have included four measures that describe evaluations of a participants ritual experience (above), and also, reports of whether the ritual has an official meaning and whether the individual knows the doctrine (below). We hypothesise that these factors will load onto one factor, a ‘cognitive’’ factor. However, in the case of the first four elements of this factor, it is possible that they will load uniquely as those questions pertain to a kind of metacognition of the ritual experience, rather than a first order evaluation. It is possible this factor does not reveal anything meaningful about the ritual *thing*, but rather about the measurement tool applied to the *thing*.

Table S11. *Ambiguous variables*

**Variable**	**Tentative Prediction**
Drug Use / Inebriation	Multiple-Cross loadings
Religiosity	Multiple-cross loadings.<br>Possibility of unique factor in alternative-n factor solutions
Memory Quality	Possible strong loadings of factors A and B.<br>Possible dominant loading on factor D.
Official Account +<br>Know meaning	May load onto a predominantly ‘cognitive factor’.<br>Possibility of unique factor in alternative-n factor solution.

Supplementary material D.

We used attention checks not as an exclusion criteria but as an indicator that the responses should be checked for the quality of responses, as previous research suggests they are an unreliable indicator of overall quality. In the US 78 (of 298) failed the single attention check, in Japan 54 (of 284) failed a single attention check, and in India 48 (of 197) failed both attention checks, and 90 failed one of the two attention checks. We examined whether those who failed the attention check varied from those who passed in systematic ways. Thus, we categorized all responses as belonging to either the imagistic or doctrinal modes per the question prompt. We conducted an ANOVA on the following key variables for each mode, within each country: *Intensity*, whether the ritual was considered *routine*, how *consequential* the ritual was to the participant, how many *times participated*, and how well the participated *remembered* the experience. We used a corrected p-value of .003 for these 15 analyses for (as there are five analyses for each 'mode' for each country).

Among US respondents, there was no significant differences in intensity (imagistic, p = .935; doctrinal, p = .470), routine (imagistic, p = .722; doctrinal, p = .507), consequentiality (imagistic, p = .480; doctrinal, p = .953), times participated (imagistic, p = .977; doctrinal, p = .196), and remember (imagistic, p = .975; doctrinal, p = .684).

Among Japanese respondents, there was no significant differences in intensity (imagistic, p = .959; doctrinal, p = .470), routine (there was an observed differences in imagistic, p = .001, but none in doctrinal, p = .275), consequentiality (imagistic, p = .669; doctrinal, p = .119), times participated (imagistic, p = .168; doctrinal, p = .755), and remember (imagistic, p = .064; doctrinal, p = .72).

Among Indian respondents, there was no significant differences in intensity (imagistic, p = .938; doctrinal, p = .393), routine (imagistic, p = .766; doctrinal, p = .669), consequentiality (imagistic, p = .397; doctrinal, p = .583), times participated (imagistic, p = .593; doctrinal, p = .608), and remember (imagistic, p = .253; doctrinal, p = .791). Given that there was no systematic pattern to the observed differences, we have opted not to exclude participants (who surpassed more basic exclusion criteria) based on failed attention checks.

Supplementary Material E.

Table S12. Full list of factor loadings

Item #	Element	Dysphoric	Euphoric	Cognitive	Frequency
3	Neg (now)	**0.84**	0.1	-0.03	0.06
2	Neg (then)	**0.84**	0.03	0.02	0.02
6	Unpleasant	**0.82**	0.08	0.05	0.02
4	Pain	**0.78**	-0.09	0.19	-0.14
12	Positive (now)	**-0.52**	0.34	0.39	-0.01
11	Positive (then)	**-0.54**	0.37	0.36	0.04
25	Inebriation	0.44	**0.65**	-0.03	-0.02
10	Enjoyable	-0.41	**0.63**	0.2	0.11
24	Substance Use	0.34	**0.63**	-0.13	-0.01
9	Exciting	-0.23	**0.62**	0.27	-0.03
20	Importance	-0.21	0	**0.76**	-0.05
19	Consequential	-0.01	-0.02	**0.72**	-0.13
5	Attention	0.19	-0.01	**0.69**	0.1
18	Reflection	0.16	0.1	**0.68**	0.07
27	Memory Quality	-0.04	0.01	**0.65**	0.24
1	Intensity	0.26	0.11	**0.58**	-0.18
16	Times Participation	-0.1	-0.17	0.02	**0.76**
15	Ritual Frequency	0.1	0.06	0	**0.75**
17	Routine	0.2	0.23	0.09	**0.62**
7	Unusual	0.31	0.08	0.17	-0.49
26	Religiousness	0.24	-0.23	0.49	0.14
13	Synchrony	0.24	0.1	0.43	-0.12
14	Sex Segregation	0.23	0.34	-0.04	0.17
8	Alone	0.22	0.23	-0.03	0.16
22	Official Meaning	0.02	-0.16	0.39	-0.08
23	Personal Meaning	-0.07	-0.18	0.38	0.04
21	Understanding	-0.22	0.13	0.45	0.07

	Proportional Variance	*0.16*	*0.09*	*0.16*	*0.08*
	Cumulative Variance	*0.16*	*0.25*	*0.41*	*0.49*
